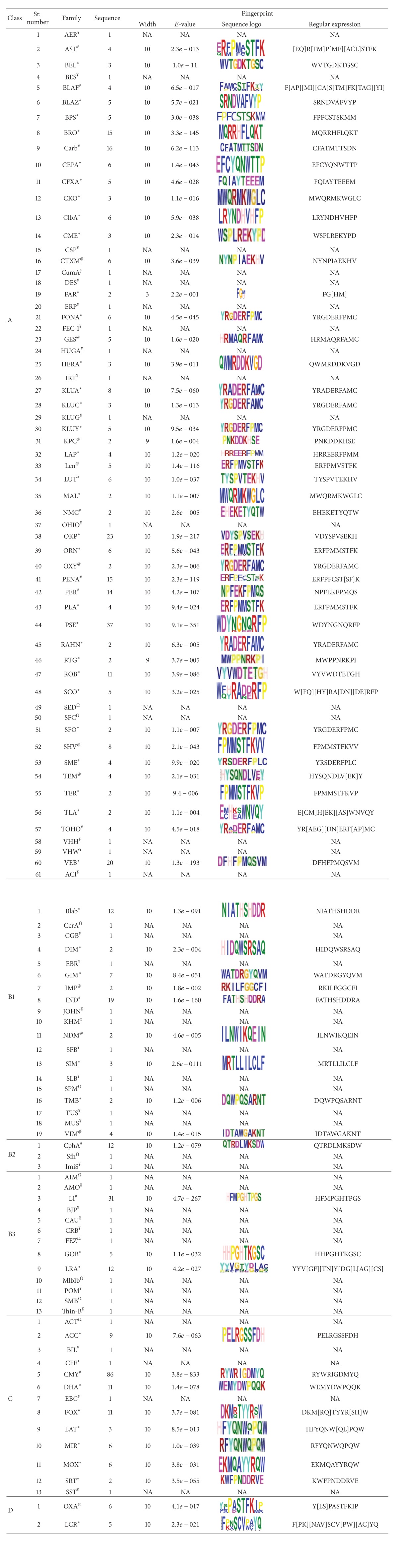# Identification of Family Specific Fingerprints in ****β****-Lactamase Families

**DOI:** 10.1155/2014/980572

**Published:** 2014-02-11

**Authors:** Abhishikha Srivastava, Neelja Singhal, Manisha Goel, Jugsharan Singh Virdi, Manish Kumar

**Affiliations:** ^1^Department of Biophysics, University of Delhi South Campus, New Delhi 110 021, India; ^2^Department of Microbiology, University of Delhi South Campus, New Delhi 110 021, India

## Abstract

Beta-lactamases are a superfamily of enzymes which degrade the **β**-lactam class of antibiotics. They are produced endogenously by the bacterial cells, which when exposed to the **β**-lactam class of antibiotics inactivate them by cleaving the **β**-lactam ring. Based on the presence or absence of metallic ligand, **β**-lactamases have been divided into two broad functional classes. **β**-Lactamases are a constitutively evolving and expanding superfamily of enzymes, which could be further subdivided on the basis of presence/absence of conserved motifs. In the present study we have used the MEME/MAST suit to identify the patterns/motifs which are specific to a particular family or subfamily of **β**-lactamases. The family specific patterns/motifs can be also useful in recognizing and assigning newly discovered **β**-lactamases to one or the other family or subfamily. Cross-validation showed that the proposed method is highly sensitive and specific. We have also designed a webserver, LactFP, for this purpose.

## 1. Introduction

Antibiotics are chemical compounds used to treat bacterial infections. The indiscriminate overuse of antibiotics leads to the evolution of antibiotic resistance in pathogenic microbes. Resistance to antibiotics can be generated by four general mechanisms [1, 2]: (i) inactivation or modification of the antibiotic; (ii) alteration in the target site of the antibiotic that reduces its binding capacity; (iii) modification of metabolic pathways to circumvent the antibiotic effect; (iv) reduced intracellular antibiotic accumulation by decreasing permeability and/or increasing active efflux of the antibiotic. *β*-Lactams, the most widely used antibiotics, are rendered ineffective by bacteria employing the first strategy, that is, cleavage of amide bond of the *β*-lactam ring.

The extensive overuse of antibiotics exerted selective pressure on bacteria, which evolved new variants of *β*-lactamases extending the *β*-lactamase family [3]. The two popular classification schemes of *β*-lactamase enzymes are Bush-Jacoby scheme which is based on functional characteristics of the enzymes [4] and Ambler's scheme [5] which is based on the amino acid sequence similarity. According to Ambler's classification scheme, *β*-lactamases are divided into four classes. Classes A, C, and D are serine-*β*-lactamases which employ an active-site serine to catalyze hydrolysis, while class B *β*-lactamases are metalloenzymes requiring one or two zinc ions for their activity.

Although “annotation transfer by homology,” which involves global comparison between two sequences, is the most popular approach for inferring function of a protein, experimental bodies of evidence suggest that protein function can be correlated well with the presence of local patterns of amino acid residues or motifs shared by proteins with similar function. Motifs are a highly conserved set of residues that form similar patterns and often represent functionally important regions such as active or binding sites, or regions defining the overall protein fold. Throughout the course of evolution, functionally important parts of proteins like active site residues in case of enzymes have remained conserved. Thus, analysis of similarity in local regions of a protein or structural motifs could be useful for predicting protein function and/or identifying functionally significant sites. This implies, that motifs can be used to assign diagnostic signatures and fingerprints to protein families based on their similarity/relatedness and assign new members to a family. Further, presence of a conserved motif can also aid in understanding the relationship of sequence and structure of a protein with its function.

Typically, sequence motifs are derived from multiple sequence alignments of proteins belonging to the same family or having similar function. There are many types of software which extract the motifs, but PROSITE patterns [6] is undoubtedly the most widely used software for mining motifs and inferring function. There are four patterns reported for *β*-lactamase family. (i) Beta-lactamase class B signatures (PDOC00606): most of them are centered on residues known [7] to be involved in binding zinc ion and essential for the enzyme's catalytic activity. There are two signature PROSITE patterns for this class of enzyme, namely, BETA_LACTAMASE_B_1 (PS00743) whose first three residues are involved in zinc ions binding and the second pattern BETA_LACTAMASE_B_2 (PS00744) which contains a cysteine and also has a zinc ligand. (ii) Beta-lactamases classes A, C, and D active site signature (PDOC00134): it contains three different types of PROSITE patterns, namely, BETA_LACTAMASE_A (PS00146), BETA_LACTAMASE_C (PS00336), and BETA_LACTAMASE_D (PS00337). Besides the PROSITE patterns, each class of *β*-lactamase can also be characterized by presence of a conserved and specific active site signature [8]. For example, sequences belonging to class A contain three conserved elements, that is, S-X-X-K, S-D-N, and K-T-G at positions 70, 130, and 234, respectively [8]. Sequence belonging to class C contains S-X-S-K, Y-S-N, and K-T-G at positions 64, 150, and 314, respectively [8]. Class D lactamase contains S-X-X-K, Y-G-N, and K-T-G at positions 70, 144, and 214, respectively [9]. Sequences belonging to class B contain H-90, D-92, L-117, H-168, G-204, and H-236 as conserved residues located at the bottom of the active site. Among these H-80, H-90, and H-168 accommodate Zn^2+^, which is required for the activity of class B *β*-lactamases [7]. The available information apparently assigns a generalized conserved pattern to each Ambler class but fails to identify family specific patterns useful for further classifying Ambler's classes into different families and subfamilies. The family specific patterns/motifs can also be used as a fingerprint to recognize new members of *β*-lactamase families.

In the present study we have used a popular motif-finding tool MEME-MAST [10] to find the characteristic motifs of each family of *β*-lactamase. To the best of our knowledge, this is a first study in which an extensive manually curated dataset of *β*-lactamase sequences was created. The whole dataset is classified into four Ambler classes, which is further divided into families. We also cross-validated the extracted motifs by carrying out search against (i) the complete lactamase dataset and (ii) UniProt protein sequence database.

## 2. Materials and Methods

### 2.1. Sequence Database Search

Initial searches were performed against UniProtKB/TrEMBL using “beta-lactamase*” and “bla*” genes as keyword. A total of 1415 protein sequences of different *β*-lactamase variants were obtained. The choice of UniProt for present work was guided by the fact that this database is regularly updated.

Initially sequences of *β*-lactamases belonging to various families were retrieved from UniProt database using “beta-lactamase*” and “bla*” genes as keyword with following criteria: (1) only proteins with *bla* gene were considered; (2) expulsion of sequence annotations containing the words “by similarity,” “probable,” or “potential”; (3) selection of experimentally existing protein sequences only. Protein sequences of *β*-lactamase genes were manually classified into families according to their source genome, hydrolyzing profile, and geographical diversity. We also carried out BLAST based similarity search with *E*-values threshold of 10^−4^ to “fish out” any missing proteins. Using the above criteria, we initially retrieved 1415 sequences and classified them into 113 individual families.

For motif finding we included only those proteins whose existence was experimentally proved. We tried to use maximum number of sequences whose experimental existence was known. Nonexperimentally existing sequences were used only in absolute necessity. In cases where *β*-lactamase families had (i) multiple proteins whose experimental existence was proved, or (ii) consisted of multiple proteins but none was experimentally proved, or (iii) comprised multiple proteins but only one member was experimentally established, all proteins were used for mining motifs. No motifs were extracted from families which had only one sequence (experimental existence proven or not). Using the above criteria we have catalogued 605 protein sequences with 325 from class A, 58 from subclass B1, 14 from subclass B2, 58 from subclass B3, 139 from class C, and 11 from class D (Table 1).

### 2.2. Conserved Motif Identification

Family specific motifs were searched using the motif discovery program MEME (ver.4.6.1) [10] (http://www.sdsc.edu/MEME), which is based on the expectation-maximization algorithm. Ungapped family specific motifs, with a maximum length of ten amino acids, were modeled using the one occurrence per sequence model (OOPS) of MEME, which represents motifs as position-dependent letter-probability matrices, which describes the probability of each possible letter at each position in the pattern. OPPS was used with the motive to find a pattern that has discriminatory power to differentiate between two families of *β*-lactamases. The OOPS model of MEME assumes that there is one occurrence of the motif in all sequences in the same family. We did not try zero or one occurrence per sequence (ZOOPS) because ZOOPS may find pattern shared among very few family members thereby not generalizing the whole data, thus compromising the discriminatory capability.

The MEME output also contains color graphical alignments as well as common regular expression of motifs along with the *E*-value, which describes the statistical significance of the motif. MEME usually finds the most statistically significant (low *E*-value) motifs first. The *E*-value is an assessment of the probable number of motifs with the given log probability ratio (or higher) along with the same width and site count present, which one would find in a similarly sized set of random sequences. Groups of position-dependent scoring matrices, as defined by MEME, were used as input to the MAST algorithm for searching a database containing sequences of all *β*-lactamases.

## 3. Results and Discussion

By default, MEME looks for up to three motifs, each of which may be present in some or all input sequences depending upon the command used while initiating the MEME search [10]. MEME chooses the width and number of occurrences of each motif automatically in order to minimize the “*E*-value” of the motif—the probability of finding an equally well-conserved pattern in random sequences. The MEME output is HTML and shows the motifs as local multiple alignments of (subsets of) the input sequences, as well as in several other formats. “Block diagrams” show the relative positions of the motifs in each of the input sequences. After the submission of sequences in query box of MEME, results are displayed in the form of graph and sequence is displayed in the form of sequence logo or regular expression (Table 1).

The presence of motifs in the majority of *β*-lactamase families indicates that the motifs are highly specific. As we had imposed maximum limit of motif as 10 residues, we later found that it served as a good cutoff. As can be seen in Table 1, in most of families the width of motif is 10 residues. In only 5 families we observed motif of less than 5 residues. When we analyzed the presence of motifs in different classes, we observed that in subclass B3, all except 3 families did not have any conserved motif. But it was not due to the absence of any conserved pattern rather it was due to presence of a single member in that family. Another interesting observation was that in majority of the sequence logo, at most positions only a single residue is present, which also points towards the nonvariable nature of the motifs. The nonvariable nature of motif constituting residues would be useful when newly discovered *β*-lactamases will be added to that family.

## 4. Cross-Validation

We adopted two approaches of cross-validation to assess the efficiency of fingerprints extracted from each *β*-lactamase family to recognize members of the same family. First approach was searching against a database that contained *β*-lactamase sequences of all family compiled together. The second approach was to and against complete UniProt database [11]. During cross-validation we took each motif library generated by MEME and searched for presence of motif using MAST. In both approaches, if the first hit (i.e., contain the same motif with minimum *E*-value) belonged to the same family, it was considered as true positive. In all families we found that the first hit always belongs to the same family. It showed that the motif library created by us is not only sensitive but also very specific.

## 5. Web-Server 

The methodology described in this paper is implemented as a web-server LactFP, which is available to the scientific community without any usage charge (http://14.139.227.92/mkumar/lactfp/). This server allows users to search the family specific fingerprint in their protein.

## 6. Conclusions

Resistance against antibiotics in pathogenic microbes is becoming a major health issue. An important cause behind antibiotic resistance is production of antibiotic degrading enzymes by the pathogens as in case of *β*-lactamases. With the discovery of a newer class of *β*-lactam antibiotics, bacteria are also evolving their *β*-lactamases giving rise to a very large divergent super-family of *β*-lactamase enzymes. Thus it becomes imperative that newly discovered *β*-lactamases are quickly identified/categorized before medical treatment could be initiated. To facilitate quick identification of *β*-lactamases, we have identified fingerprints which are unique characteristic to a specific family. This catalogue is based on a dataset of 605 manually curated *β*-lactamase enzymes. We have also verified the efficiency of fingerprints in finding the family of new *β*-lactamase sequence using UniProt protein database and all 605 *β*-lactamase sequences. The results show that the catalogued fingerprints can predict the family with very high specificity.

## Figures and Tables

**Table 1:** List of fingerprints in different families of *β*-lactamase. (^@^fingerprint is extracted from more than one sequence with evidence at protein existence level; ^#^only one sequence has evidence at protein existence level, and hence proteins whose existence is not experimentally proven were also selected; *only sequences without evidence of protein existence were present in the family, and hence fingerprint is based on nonexperimentally existence proteins; ^Ω^only one sequence is present in the family with evidence of protein existence; ^¥^only one sequence is present in the family whose existence is not proven at protein level).